# The Story of the Insane from Year to Year

**Published:** 1901-12-28

**Authors:** 


### THE STORY OF THE INSANE FROM YEAR TO YEAR.

(THIRTEENTH SERIES.)

### THE WARNEFORD ASYLUM, OXFORD.

THIS charitable hospital for the insane contained 93 patients on January 1st, 1899. There were 14 first admissions, 1 re-admission, 8 recoveries, 1 discharged relieved, and 5 deaths. The average daily number resident during the year was 91, and the total number of persons under treatment was 108. Calculated on the admissions, the recoveries stand at 72·7 per cent., and calculated on the average number resident the deaths stand at 5·5 per cent. The percentage of recoveries is very high; but, as we have elsewhere said, no very definite conclusion can be drawn from this fact when the numbers dealt with are so low. In fact, last year the recovery rate was 16·6 per cent., and for a period of 54 years it shows the fair average of 37·6 per cent. Cerebral and spinal diseases account for 3 of the deaths, pneumonia for 1, and senile decay for 1. It is admitted that this is one of the hospitals which is still conducted on the charitable lines for which it came into existence, and the following extract from Dr. Neil's report shows not only this fact, but also how two others of hese so-called charitable institutions discharged their obligations. "A young man, the son of a clergyman and in training for Holy Orders, broke down mentally. He was treated for a time in one of the registered hospitals, whence he was removed for pecuniary reasons to another, where the charge was somewhat less. His friends soon found it necessary to petition the committee of this hospital for a reduction of payment. The petition was not successful, and the patient finally landed here, where in all probability he will be kept, and kept in comfort for the rest of his life." Dr. Neil does not name the two hospitals who turned this unfortunate patient out; but certainly they ought to be exposed, for had it not been for Warneford, which is very probably less rich than either of the others, the patient would inevitably have drifted to the County Asylum. This is far from being an isolated instance. On page 24 of this well-printed and well-illustrated report we get a table which we have often insisted ought to be published by all these lunatic hospitals; but some of them evidently dread publicity. We notice that 1 patient paid 5s. 9d. a week; 1 paid 6s. 6d.; 2 paid 8s.; 17 paid 10s.; and not fewer than 45 others paid less—some of them much less—than the actual weekly cost per head, which was 28s. 1d. It is a sad reflection that this hospital and a few others we could name are crippled by want of accommodation. A general infirmary has no difficulty in raising almost unlimited capital for extensions; but the very name of "lunatic" seems to scare capitalists. Why should not Warneford be provided with another 50 beds which would enable it to extend its good work? The asylum is evidently well managed by Dr. Neil; but we hear that one poor lady complained to the Commissioners in Lunacy that she was not allowed to crochet on Sundays. Nothing is more certain than that this patient would equally have complained had she been asked to crochet on Sunday; but all the same we agree with the commissioners that it would be better to work than sit with folded hands "in listless apathy." From the report of the committee we learn that they are opposed to the clause in the ill-fated Lunacy Acts Amendment Bill relating to lunatic hospitals. We think the committee are fully justified in this opposition, and we hope their opposition will be taken up by other committees, and that it will be in the end successful. The committee refer to "the satisfactory state of the asylum under Dr. Neil's direction."

